# Membranous nephropathy with acquired factor V inhibitor: a case report

**DOI:** 10.1186/1756-0500-6-553

**Published:** 2013-12-21

**Authors:** Shinji Kitamura, Mahito Misawa, Sayaka Namba, Kenji Tsuji, Rikako Torigoe, Midori Shima, Hirofumi Makino

**Affiliations:** 1 Department of Medicine and Clinical Science, Okayama University Graduate School of Medicine, Dentistry and Pharmaceutical Sciences, 2-5-1 Shikata-cho, Okayama 700-8558, Japan; 2 Department of Internal Medicine, Ako Central Hospital, Ako, Japan; 3 Department of dermatology, Ako Central Hospital, Ako, Japan; 4 Department of Pediatrics, Nara Medical University, Kashihara, Japan

## Abstract

**Background:**

Membranous nephropathy is one of the most common causes of nephrotic syndrome in adults. In contrast, acquired factor V inhibitor is a rare bleeding disorder.

**Case presentation:**

A 62-year-old Asian man with a history of cerebral hemorrhage, purpura, eosinophilia and hyper immunoglobulin E syndrome developed proteinuria. The bleeding disorder was diagnosed with acquired factor V inhibitors. A renal biopsy revealed that he suffered from membranous nephropathy with glomerular endothelial damage which is reported to be involved in another factor disorder. After the steroid administration, the coagulation test and proteinuria were improved.

**Conclusions:**

The presence of factor V inhibitors may have been involved in the development of membranous nephropathy.

## Background

Membranous nephropathy (MN) is one of the most common causes of nephrotic syndrome in adults. MN occurs as a primary or secondary renal disease. Secondary MN occurs as a result of autoimmune diseases, infections and malignancies. In contrast, acquired factor V (FV) inhibitor is a rare bleeding disorder that is known to be difficult for physicians to treat because of limited knowledge and its uncertain relationship with autoimmune disease. Here we suggest a relationship between MN and FV inhibitors.

## Case presentation

**Figure 1:**
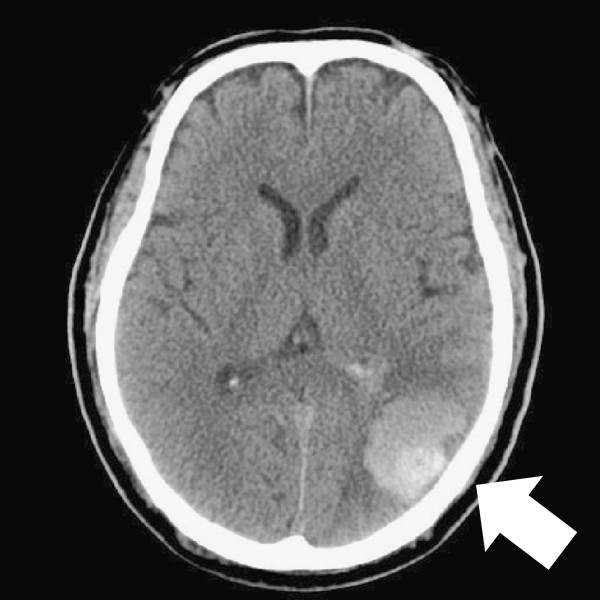
Left cerebral hemorrhage (arrow) image on computerized tomography.

**Figure 2:**
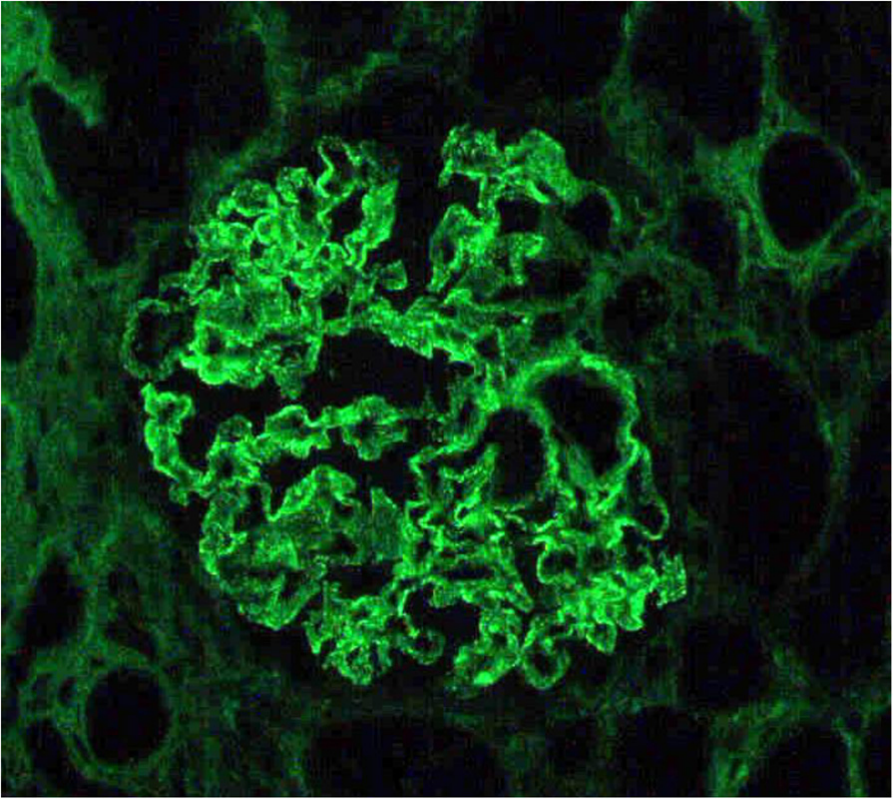
**Immunoglobulin G fluorescent staining analysis revealed that fine granular depositions were observed at subepithelial layer in glomerulus.** (Magnification: 400X).

**Figure 3:**
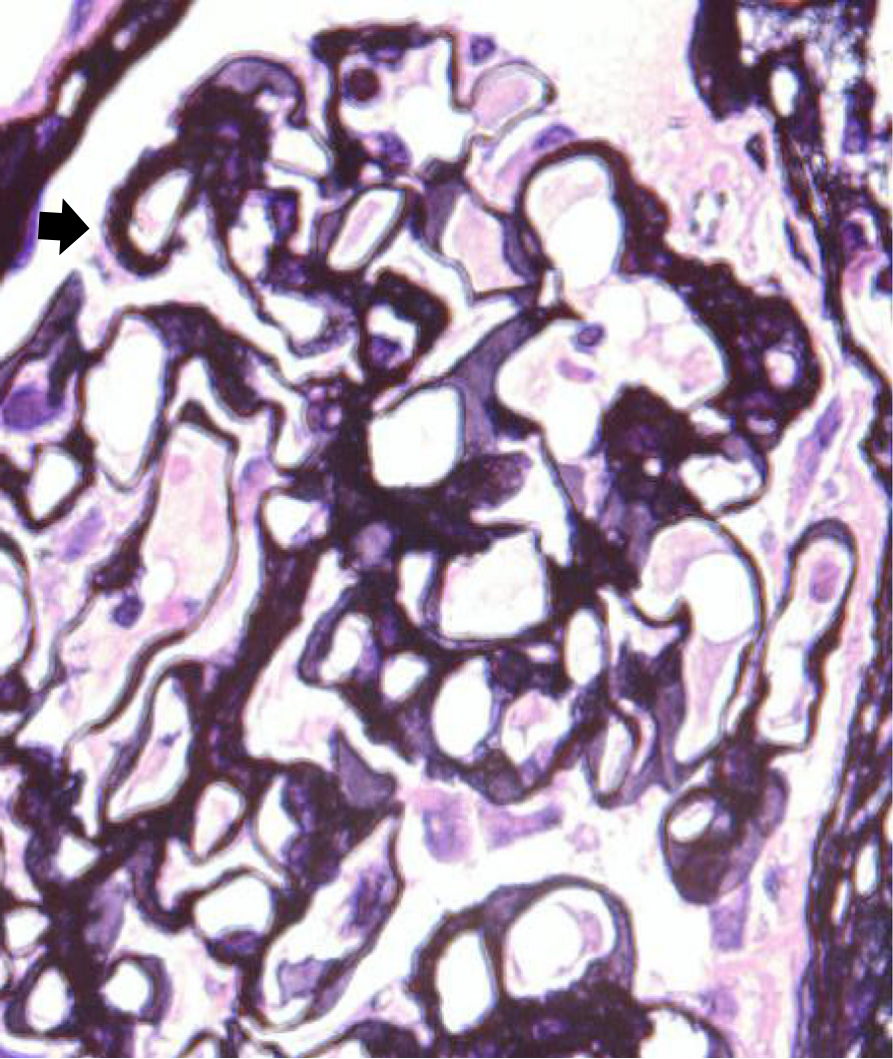
**Periodic acid-methenamine-silver staining analysis showed that spike formations (arrow) were observed partially at the subepithelial layer in the glomerulus.** (Magnification: 400X).

**Figure 4:**
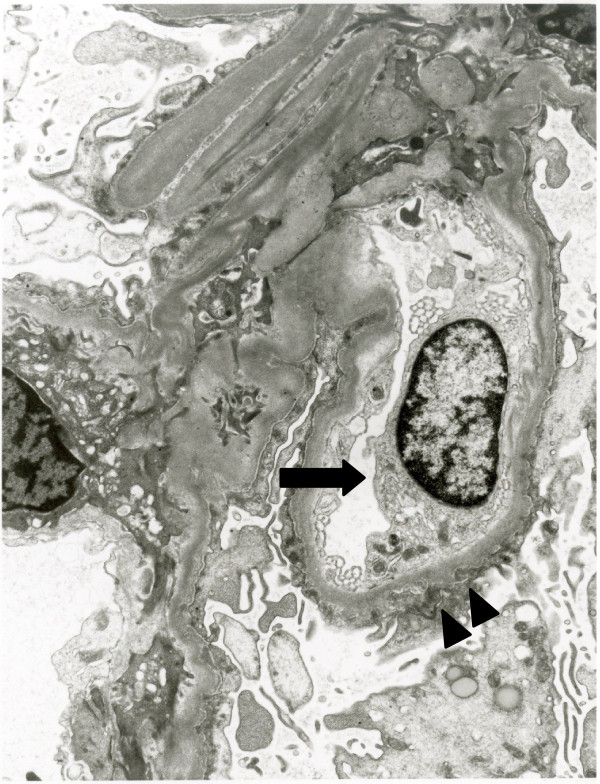
**Electron microscopic analysis demonstrated the swelling of the endothelial cell (arrow), the damage of lamina rara layer and electron dense deposit at the subepithelium (arrow head).** (Magnification: 8050X).

A 62-year-old Asian man consulted a doctor because of asthmatoid wheeze, anarthria, purpura and gait disturbance. He has no history of hypertension. He pointed out proteinuria for the first time two months ago before the consultation. He was diagnosed with a cerebral hemorrhage following a computerized tomography scan (Figure 1). His laboratory findings revealed that his serum creatinine concentration was 0.66 mg/dl, his serum IgE concentration was 18230 IU/ml (normal: <170 IU/ml), and his eosinophil count was 18900/μl. His urinary analysis revealed 1.61 g/gCr of proteinuria. Coagulation tests revealed a prolonged activated partial thromboplastin time at 61.2 seconds and a prothrombin time of 25.5 seconds. In addition, FV activity alone severely decreased to 4.4% of normal, and an FV inhibitor was present at a titer of 2.5 BU/ml, suggesting the presence of antibody-mediated circulating inhibitors specific for FV (Table 1). The patient was diagnosed with a cerebral hemorrhage, eosinophilia, hyper IgE syndrome and acquired FV inhibitors. Steroid therapy with prednisolone (1 mg/kg) for the treatment of purpura and acquired FV inhibitors was administered. Treatment with steroid led to the improvement of his clinical symptoms including purpura, normalization of the coagulation tests, and disappearance of eosinophilia. To confirm the diagnosis of proteinuria, we performed a renal biopsy. Fine granular depositions were observed at the subepithelial layer in the glomerulus upon IgG fluorescent staining (Figure 2). Spike formations were partially observed at the subepithelial layer upon Periodic acid-methenamine-silver (PAM) staining (Figure 3). An impaired lamina rara layer and endothelial cell swelling and detachment were observed with high-density deposits at the lamina rara externa upon electron microscopic analysis (Figure 4, Additional file 1: Figure S1 and Additional file 2: Figure S2). We determined that the patient had developed MN with glomerular endothelial cell damage. After the administration of steroid therapy, the proteinuria improved gradually.

**Table 1:** Laboratory analysis data of coagulation time and coagulation factors

**Coagulation time**	**Time (sec)**
Prothrombin time	25.5
Activated partial thromboplastin time	61.2
Coagulation factors	Activity (%)
Factor II	93.0
Factor V	4.4
Factor VIII	77.0
Factor IX	168.4
Factor X	93.0

## Discussion

MN is caused by immune complex localization in the subepithelial zone of glomerular capillaries. Beck *et al.* reported that M-type phospholipase A_2_ receptor (PLA_2_R) is a target antigen with idiopathic MN [1]. The Anti- PLA_2_R autoantibodies in serum samples from patients with idiopathic MN were predominantly of IgG4 subclass, which is the predominant immunoglobulin subclass seen in glomerular deposits of patients with MN. However, the Anti- PLA_2_R autoantibodies were not exclusively found in secondary MN. In renal biopsy of this patient, we could not observe the deposits of IgG4 subclass (Additional file 3: Figure S3). We could not find any causes of secondary MN such as malignancy, infections or drugs. These results suggested that the MN in this patient may be involved other immune disorders.

On the other hand, there have been some reports of acquired factor inhibitors complicated by nephrotic syndrome [2-4]. In addition, there have been reports that factor VIII-related antigen and tissue plasminogen activator may be involved in the glomerular endothelial damage in another factor disorder [5]. There is a possibility that coagulopathies may be related to the occurrence of renal disorders with glomerular endothelial cell damages. In this patient, we observed dense subepithelial deposition and the detachment of endothelial cells in the glomerulus upon electron microscopic analysis. It may be suggested that the characteristic finding of membranous nephropathy with acquired factor inhibitors is the presence of damage of the glomerular endothelial cells and lamina rara layer, with high-density deposits at the subepithelium in the glomerulus. Frigui *et al*. reported that the eosinophils are associated with MN [6]. Renal disorders with eosinophils were caused by several diseases such as drugs, cholesterol embolization, immunoallergic responses et al. [7]. Giudicelli *et al.* described that most secondary renal disorders in hyperphils are usually due to an immuno-allergic process leading to deposit of immune complexes in glomeruli. Taken together, we reported that membranous nephropathy complicated with factor V inhibitor. The presence of FV inhibitors should be taken into consideration when patients are diagnosed with nephrotic syndrome and MN by renal biopsy because there is some possibility that the FV inhibitors can lead to serious bleeding complications.

## Conclusions

We reported a case of membranous nephropathy with acquired factor V inhibitor. The renal pathology revealed the presence of damage of the glomerular endothelial cells and lamina rara layer, with high-density deposits at the subepithelium in the glomerulus. It is important that membranous nephropathy may be complicated with acquired factor V inhibitor if the pathology is observed.

## Consent

Written informed consent was obtained from the patient for publication of this Case Report and any accompanying images. A copy of the written consent is available for review by the Editor-in-chief of this journal.

## Competing interests

The authors declare that they have no competing interests.

## Authors’ contribution

SK, MM, RT and SN were the treating physicians of the patient. MS evaluated the laboratory analysis data of coagulation time and coagulation factors. SK, SN, KT and HM performed the evaluation of the renal biopsy. All authors participated in the discussion of the manuscript and approved the final version.

## Supplementary Material

Additional file 1: Figure S1 Electron microscopic analysis demonstrating the detachment of endothelial cells (arrowhead), damage to the lamina rara layer and electron-dense deposits at the subepithelium (arrow). (Magnification: 8050X). Click here for file

Additional file 2: Figure S2 Electron microscopic analysis demonstrating the electrondense deposits at the subepithelium (arrow). (Magnification: 6900X). Click here for file

Additional file 3: Figure S3 Immunohistochemistry analysis demonstrating not for the immunoglobulin G4 deposits but for immunoglobulin G deposits in the glomeruli of the patient. deposits at the subepithelium (arrow). (Magnification: 400X). Click here for file

## References

[B1] Beck LH Jr Bonegio RG Lambeau G Beck DM Powell DW Cummins TD Klein JB Salant DJ M-type phospholipase A2 receptor as target antigen in idiopathic membranous nephropathy N Engl J Med 2009 6 11 21 10.1056/NEJMoa0810457 19571279 PMC2762083

[B2] Takahashi H Fuse I Abe T Yoshino N Aizawa Y Acquired factor V inhibitor complicated by Hashimoto’s thyroditis, primary biliary cirrhosis and membranous nephropathy Blood Coagul Fibrinolysis 2003 6 87 93 10.1097/00001721-200301000-00016 12544735

[B3] Verghese P Darrow S Kurth MH Reed RC Kim Y Kearney S Successful management of factor IX inhibitor-associated nephrotic syndrome in a hemophilia B patient Pediatr Nephrol 2013 6 823 826 10.1007/s00467-012-2397-0 23381011

[B4] Chang H Chen YM Dunn P Factor VIII inhibitor associated with nephrotic syndrome Haemophilia 2007 6 766 10.1111/j.1365-2516.2007.01467.x 17973855

[B5] Imai C Kakihara T Iwabuchi H Tanaka A Furukawa T Uchiyama M Glomerular vasculopathy after unrelated cord blood transplantation Pediatr Nephrol 2003 6 399 402 12700971 10.1007/s00467-003-1081-9

[B6] Frigui M Hmida MB Jallouli M Kechaou M Frikha F Bahloul Z Membranous glomerulopathy associated with idiopathic hypereosinophilic syndrome Saudi J Kidney Dis Transpl 2010 6 320 322 20228521

[B7] Giudicelli CP Didelot F Duvic C Desrame J Herody M Nedelec G Eosinophilia and renal pathology Med Trop (Mars) 1998 6 477 481 10410369

