# Supplementary figures and images for: Membranous nephropathy with acquired factor V inhibitor: a case report
Source: BMC Res Notes. 2013 Dec 21;6:553. doi: 10.1186/1756-0500-6-553 (PMC3892019; doi:10.1186/1756-0500-6-553)

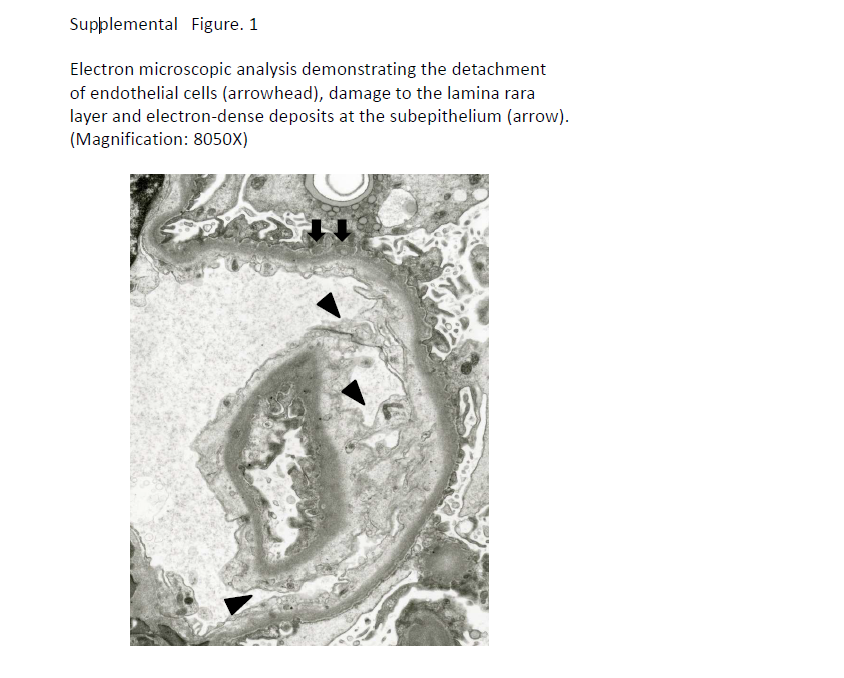

Supplement: Additional file 1: Figure S1 — Electron microscopic analysis demonstrating the detachment of endothelial cells (arrowhead), damage to the lamina rara layer and electron-dense deposits at the subepithelium (arrow). (Magnification: 8050X). [file 1756-0500-6-553-S1.bmp]

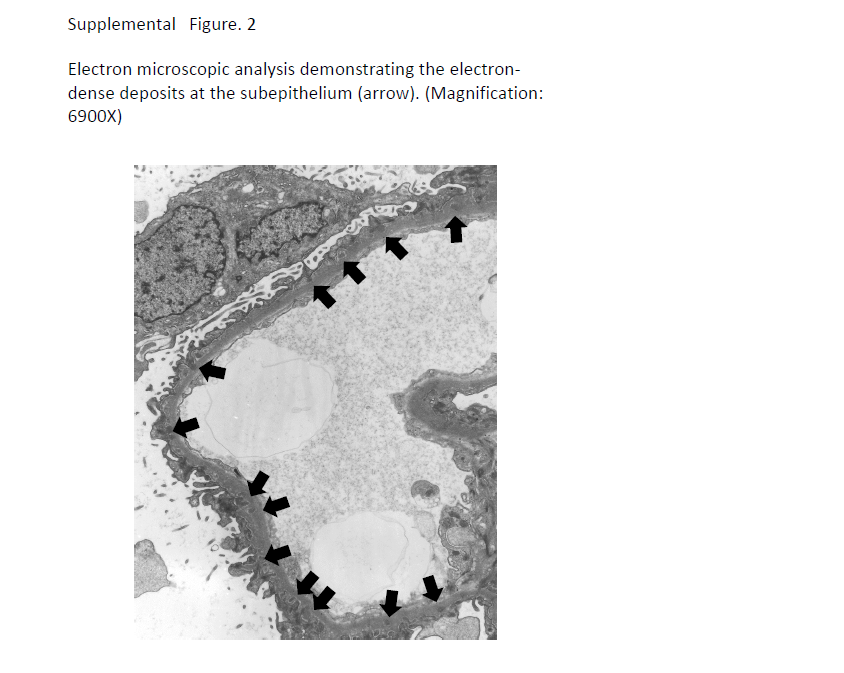

Supplement: Additional file 2: Figure S2 — Electron microscopic analysis demonstrating the electrondense deposits at the subepithelium (arrow). (Magnification: 6900X). [file 1756-0500-6-553-S2.bmp]

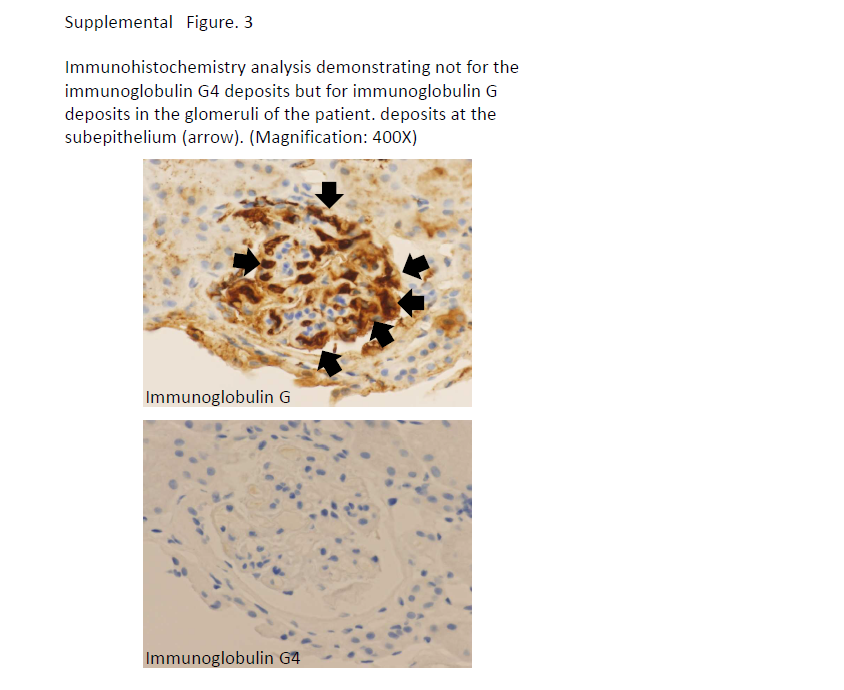

Supplement: Additional file 3: Figure S3 — Immunohistochemistry analysis demonstrating not for the immunoglobulin G4 deposits but for immunoglobulin G deposits in the glomeruli of the patient. deposits at the subepithelium (arrow). (Magnification: 400X). [file 1756-0500-6-553-S3.bmp]
